# Neighborhood’s locality, road types, and residents’ multimorbidity: evidence from China’s middle-aged and older adults

**DOI:** 10.1186/s12889-020-09876-y

**Published:** 2020-11-16

**Authors:** Xuexin Yu, Wei Zhang

**Affiliations:** West China Biomedical Big Data Center, West China Hospital, Sichuan University, No.37 Guoxue Alley, Chengdu, 610040 Sichuan China

**Keywords:** Non-communicable chronic disease, Multimorbidity, Neighborhood, Walkability, Urban-rural disparities, Middle-aged, Older adults

## Abstract

**Background:**

Neighborhood factors have gained increasing attention, while the association between the neighborhood’s characteristics and multimorbidity has not been clarified. In this study, we aim to depict variations in the number of non-communicable chronic diseases (NCDs) as a function of urban vs. rural settings and road types.

**Methods:**

The present cross-sectional study derived data from the China Health and Retirement Longitudinal Study 2011 National Baseline Survey. Negative binomial regression with clustered robust standard errors was performed to analyze variations in the number of NCDs among 13,414 Chinese middle-aged and older adults. Logistic regression models were employed to investigate the association between neighborhood-level characteristics and each NCD, respectively.

**Results:**

First, over 65% of subjects had at least one NCDs, and over 35% had multimorbidity. Arthritis (33.08%), hypertension (24.54%), and digestive disease (21.98%) were the most prevalent NCDs. Urban vs. rural differences in multimorbidity were fully explained by neighborhood clustering variations (IRR = 1.02, 95% CI, 0.95–1.10). Living with paved roads was associated with a smaller number of NCDs relative to living with unpaved roads (IRR = 0.86, 95% CI, 0.78–0.95). Results from subgroup analyses suggested that in comparison with those living with unpaved roads, individuals living with paved roads respectively had lower odds of chronic lung disease (OR = 0.76, 95% CI, 0.63–0.93), chronic liver disease (OR = 0.74, 95% CI, 0.55–0.99), chronic kidney disease (OR = 0.68, 95% CI, 0.51–0.89), digestive disease (OR = 0.82, 95% CI, 0.69–0.97), arthritis or rheumatism (OR = 0.69, 95% CI, 0.55–0.87), and asthma (OR = 0.67, 95% CI, 0.51–0.88).

**Conclusions:**

Urban vs. rural disparities in multimorbidity appeared to result from within-neighborhoods characteristics. The improvement in neighborhood-level characteristics, such as road pavement, holds promise to alleviate the increasing disease burden of chronic diseases.

## Background

Multimorbidity refers to the coexistence of two or more chronic diseases and has gained increasing attention in the context of population aging [1]. Relative to the younger adults, older adults are more likely to have chronic kidney disease [2], stroke [3], and heart disease [4], which are all major causes of deaths of older adults [5]. What’s more, patients with multimorbidity have a higher probability of premature death [6], longer hospital length of stay [7], functional disability [8], and mental problems [9]. Care for patients with multimorbidity is challenging since most of the current clinical guidelines are specific for single chronic diseases [10, 11].

The effect of neighborhood factors has been well documented in western countries, along with an increasing number of studies from eastern countries such as China [12–14]. In the context of rapid urbanization, China’s urban areas have accommodated an unprecedented number of citizens. Prior investigators have noted the linkage between neighborhood characteristics (floor-area ratio, building density, mixed land use, and green space) and residents’ mental health in urban China [15, 16]. Liu et al. (2018) noted that the neighborhood’s built, natural, and social environment was positively associated with Chinese’s self-rated health [17]. Luo et al. (2019) found that neighborhoods’ basic infrastructures, bus lines, and handicap access were associated with residents’ cognitive function [18]. However, limited attention has been directed to the association between the neighborhood’s characteristics and multimorbidity.

### Neighborhood’s locality

Despite that extensive studies have focused on the urban vs. rural disparities in multimorbidity, the vast majority of existing research did not control for neighborhood-level variations [19–23]. For example, there are two relevant studies based on the Chinese population, the results of which suggest that urban residents have higher odds for multimorbidity [21, 24]. Both studies, however, employed between-person analyses, which did not allow them to adjust for neighborhood-level variations and any neighborhood characteristics [21, 24]. Whether the urban-rural disparities in multimorbidity remain after controlling for neighborhood-level variations is unknown.

### Neighborhood’s road types

Living in walkable neighborhoods has been recognized as a protective factor of residents’ health. For example, living in a neighborhood with access to greenness and open space may provide residents a space for walking and social coherence, and therefore lead to better mental and physical health [25, 26]. The effect of living in a walkable neighborhood on residents’ health as a reflection of non-communicable chronic disease is worthy of exploration, particularly in China, where the vast majority of individuals in China utilize in-center health services. In addition to the effect on social coherence and recreation [25, 26], living in a walkable neighborhood could play a key role in residents’ access to health care and ultimately affect the individuals’ regular and long-term chronic disease.

However, little research has investigated the association between walkable neighborhoods and multimorbidity in China, for which the adoption of walkability measures is a major issue. Prior studies have defined the extent to which the neighborhood’s built environment affects walking as walkability [27]. Walkability incorporates a variety of measures including but not limited to land use mix [28, 29], residential density [28, 29], access to recreational and green space [25], access to transportation [30, 31], and street connectivity [32]. Various indicators of walkability have been proposed, including subjective perception and objective measures that employed individual surveys or GIS-based approaches [27, 29, 33–35]. However, it is well-acknowledged that a “one-size-fits-all” indicator of walkability does not exist. Although a prior study has modified and validated Neighborhood Environment Walkability Scale-Abbreviated (NEWS-A) among Cantonese-speaking older adults in Hongkong, the culture and city planning differ substantially between Hongkong and other cities in China [33]. The validated version should be cautiously administered in mainland China before further modification and validation. Moreover, the existing measurements that developed in urban cities may not be applied to rural China, where well-defined aspects of walkability, such as access to recreational space and supermarket, maybe inappropriately examined because of the inadequate facilities.

The present study proposed road types as a potential indicator of the neighborhood’s walkability for the following reasons. China has launched several ambitious plans to improve rural infrastructure, of which investment in rural roads dominated a considerable share [36]. Although the expansion of rural roads has linked rural residents to the outside world, where job opportunities, medical services, and schools are, the quality of roads has raised serious concerns [37, 38]. Upgraded or paved roads may decrease time costs for access to health services, particularly in less developed countries where the rainy season would render unsurfaced roads impassable [38–40]. Given that the association between access to health care and the population’s well-being has been received [41–43], and there is initial evidence linking road constructions with residents’ cognition [18], living with paved roads may be associated with residents’ multimorbidity, which requires substantial regular and long-term health care services [22, 44]. However, there exists limited evidence directly linking neighborhoods’ road types with multimorbidity, and the present study aims to address the gap.

### Objectives and hypotheses

Overall, this study aims to investigate the association between two neighborhood characteristics and multimorbidity among Chinese middle-aged and older adults. We offered two hypotheses for data analyses. First, urban-rural disparities in multimorbidity may not be consistent after adjusting for neighborhood-level variations (H_1_). Second, living in a walkable neighborhood may be associated with fewer NCDs (H_2_) since walkable neighborhoods could increase residents’ access to health care [45]. Here, we measured road quality by differentiating roads into paved roads vs. unpaved roads to reflect the neighborhood’s walkability. We defined the paved road as a more walkable road compared with the unpaved road. However, cautiousness should be taken when generalizing our findings into the literature of walkability in relation to health.

## Methods

### Data source, study design, and study sample

We employed a cross-sectional study. Data came from the China Health and Retirement Longitudinal Study (CHARLS) 2011 National Baseline Survey, which included 17,708 Chinese aged over 45 [46]. Individuals came from 28 provinces in mainland China and were recruited by a multistage random sampling procedure [46]. Information regarding sampling, recruitment, response rate, and procedures for data collection could be retrieved from a prior study [46]. The present study used individual-level data from the individual survey as well as neighborhood-level characteristics from the community questionnaire from CHARLS. In our definition, neighborhoods refer to villages in rural areas and communities in urban areas. As CHARLS was a community-based survey, individuals were clustered within their neighborhoods (communities or villages) by sharing the unique neighborhood identification. Excluding 4295 observations with missing data, we derived data on 13,413 subjects from 432 neighborhoods.

### Measures

#### Outcomes

Whether a subject had ever been diagnosed with the fourteen non-communicable chronic diseases was recorded in the survey. The fourteen non-communicable chronic diseases included hypertension, dyslipidemia, diabetes, cancer or malignant tumor, chronic lung diseases, chronic liver disease, heart problems, stroke, chronic kidney disease, stomach or other digestive diseases, mental problems, memory-related disease, arthritis or rheumatism, and asthma. Data were derived from answers to the question, *“Have you been diagnosed with the following 14 NCDs?”.* In addition, the data on hypertension, chronic lung disease, and mental problems also included answers to the question: “*Do you know if you have hypertension, chronic lung disease, and mental problems, respectively?”*

Multimorbidity is the primary outcome identified as the overall number of non-communicable chronic diseases. The values of multimorbidity ranged from 0 to 14. Additionally, we respectively identified the diagnosis of the 14 NCDs as the secondary outcomes (1 vs. 0).

#### Exposures

The first exposure is the urban versus rural settings to reflect neighborhoods’ locality. Second, we focused on the neighborhood’s road types and categorized roads into unpaved roads, paved roads, and others (e.g., sand-stone roads and highways). Data about road types came from the question *“What type of road does your village/community mainly have?”* in CHARLS’s community questionnaire.

#### Neighborhood-level covariates

The number of primary care institutions within the neighborhood (community health centers, community health care medical posts, township health clinics, and village medical posts) was obtained to measure residents’ access to primary care since prior studies have documented that access to health care resources could be associated with population health [41–43]. We categorized the number of primary care institutions in each neighborhood (village or community) into 0, 1, 2, and ≥ 3. Data were derived from the question “*How many community health centers, community health care medical posts, township health clinics, or village medical posts in the village or community?”.* Last, whether the neighborhood has the groundwater system or not (yes vs. no) was introduced as a covariate to reflect the neighborhood’s living conditions.

#### Individual-level covariates

Individual-level confounders including age (in a unit of years), sex (women vs. men), marital status (married vs. others), education attainment (illiteracy, some primary school, primary school, and junior school or above), household income (1st, 2nd, 3rd, and 4th), body mass index (BMI, < 18.5, ≥18.5 and < 25, ≥25 and < 30, and ≥ 30), physical activity (never, seldom, on a weekly basis, and on a daily basis), and health care insurance were introduced as covariates since prior observations have considered them to be potential indicators of multimorbidity [6, 9, 47–50]. Health care insurance was classified as uninsured, rural cooperative medical insurance (RCMI), and others, which included business medical insurance, Urban Residents Medical Insurance, and Urban Employees Medical Insurance, due to a limited number of subjects with the last three types of medical insurance in CHARLS.

### Statistical analyses

First, we stratified study subjects into urban and rural groups to exam the distribution of the baseline characteristics. T-test was employed for age. Mann–Whitney U tests were employed for the number of primary care institutions, educational attainment, household income, BMI, and physical activity. Chi-squared tests were employed for road types, groundwater systems, sex, marital status, and health care insurance. The analyses were used for in-sample interpretations; thereby, CHARLS survey weights were not introduced (Table 1).

**Table 1:** Baseline characteristics of study sample, stratified by urban vs rural settings, n (%)

Characteristic	Total (*n* = 13,413)	Urban (*n* = 5639)	Rural (*n* = 7774)	*P* value
Road type				
unpaved roads	3104 (23.14)	1001 (17.75)	2103 (27.05)	< 0.0001
paved roads	8767 (65.36)	4173 (74.00)	4594 (59.09)	
others	1541 (11.50)	465 (8.54)	1077 (13.85)	
Primary care institutions				
0	3226 (24.05)	1333 (23.64)	1893 (24.35)	0.07
1	5188 (38.68)	2210 (39.19)	2978 (38.31)	
2	3098 (23.10)	1339 (23.75)	1759 (22.63)	
≥ 3	1901 (14.17)	757 (13.42)	1144 (14.72)	
Groundwater system				
no	9495 (70.79)	3290 (58.34)	6205 (79.82)	< 0.0001
yes	3918 (29.21)	2349 (41.66)	1569 (20.18)	
Sex				
men	6431 (47.95)	2660 (47.17)	3771 (48.51)	0.13
women	6982 (52.05)	2979 (52.83)	4003 (51.49)	
Age, years ^a^	59.09 (±10.16)	58.91 (±10.14)	(59.21 (±10.18)	< 0.0001
Marital status				
married	10,741 (80.08)	4475 (79.36)	6266 (80.60)	0.08
other	2672 (19.92)	1164 (20.64)	1508 (19.40)	
Education attainment				
illiteracy	3672 (27.38)	1543 (27.36)	2129 (27.39)	0.67
some primary school	2371 (17.68)	1019 (18.07)	1352 (17.39)	
primary school	2895 (21.58)	1195 (21.19)	1700 (21.87)	
junior school or above	4475 (33.36)	1882 (33.37)	2593 (33.35)	
Household income (quartile)				
1st (the poorest)	3218 (23.99)	1477 (26.19)	1741 (22.40)	< 0.0001
2nd	3466 (25.84)	1363 (24.17)	2103 (27.05)	
3rd	3345 (24.94)	1418 (25.15)	1927 (24.79)	
4th (the richest)	3384 (25.23)	1381 (24.49)	2003 (25.77)	
BMI (kg/m^2^)				
< 18.5	935 (6.97)	358 (6.35)	577 (7.42)	< 0.001
≥ 18.5 and < 25	8362 (62.34)	3449 (61.16)	4913 (63.20)	
≥ 25 and < 30 (overweight)	3462 (25.81)	1532 (27.17)	1930 (24.83)	
≥ 30 (obese)	654 (4.88)	300 (5.32)	354 (4.55)	
Health care insurance				
uninsured	893 (6.66)	375 (6.65)	518 (6.66)	0.14
RCMI	9771 (72.85)	4063 (72.05)	570 (73.42)	
others	2749 (20.50)	1201 (21.30)	1548 (19.91)	
Physical activity				
never	12,686 (94.58)	5144 (91.22)	7542 (97.02)	< 0.0001
seldom	112 (0.84)	75 (1.33)	37 (0.48)	
a weekly basis	129 (0.96)	88 (1.56)	41 (0.53)	
a daily basis	486 (3.62)	332 (5.89)	154 (1.98)	

Note: ^a^ mean (± standard deviation)

When estimating the prevalence of multimorbidity and each NCD among China’s middle-aged and older adults, CHARLS complex sampling weights were used to account for selection bias (Table 2). Since there were no significant differences between results from weighted analyses and unweighted analyses, unweighted analyses were used for the following modeling analysis.

**Table 2:** Weighted and unweighted estimation of the prevalence of multimorbidity and fourteen NCDs by urban vs. rural areas in 2011

Characteristics	Unweighted sample, n (%)			Weighted to reflect to China’s population, % (95 CI)					
	All (*N* = 13,413)	Urban (*N* = 5639)	Rural (*N* = 7774)	All		Urban		Rural	
Chronic disease									
0	4216 (31.43)	1730 (30.68)	2486 (31.98)	32.09	(28.97–34.11)	32.61	(30.62–34.67)	31.48	(30.50–33.72)
1	4020 (29.97)	1665 (29.53)	2355 (30.29)	30.58	(28.73–32.57)	30.55	(29.25–31.89)	30.62	(29.46–31.73)
2	2648 (19.74)	1146 (20.32)	1502 (19.32)	19.11	(17.89–21.28)	18.75	(17.65–19.90)	19.53	(18.14–20.12)
3	1393 (10.39)	586 (10.39)	807 (10.38)	9.81	(8.58–10.62)	10.04	(9.19–10.95)	9.55	(9.16–10.50)
4	680 (5.07)	310 (5.5)	370 (4.76)	4.82	(4.17–6.07)	4.64	(4.08–5.28)	5.04	(4.31–5.39)
5	304 (2.27)	131 (2.32)	173 (2.23)	2.25	(1.61–2.72)	2.39	(1.95–2.92)	2.10	(1.92–2.65)
6	97 (0.72)	46 (0.82)	51 (0.66)	0.93	(0.69–2.25)	0.65	(0.47–0.91)	1.25	(0.62–1.38)
7	37 (0.28)	17 (0.3)	20 (0.26)	0.27	(0.17–0.54)	0.24	(0.14–0.42)	0.31	(0.18–0.40)
8	12 (0.09)	5 (0.09)	7 (0.09)	0.09	(0.03–0.21)	0.09	(0.04–0.22)	0.08	(0.05–0.16)
9	4 (0.03)	2 (0.04)	2 (0.03)	0.03	(0.01–0.12)	0.03	(0.01–0.12)	0.03	(0.01–0.08)
10	2 (0.01)	1 (0.02)	1 (0.01)	0.02	(0.00–0.16)	0.01	(0.00–0.08)	0.02	(0.00–0.07)
arthritis or rheumatism	4570 (34.07)	1923 (34.1)	2647 (34.05)	33.08	(31.29–34.93)	32.87	(30.59–35.24)	33.33	(30.53–36.25)
hypertension	3276 (24.42)	1453 (25.77)	1823 (23.45)	24.54	(22.85–26.31)	23.69	(21.91–25.58)	25.53	(22.55–28.75)
digestive disease	3083 (22.99)	1251 (22.18)	1832 (23.57)	21.98	(20.68–23.33)	22.69	(21.24–24.21)	21.15	(18.92–23.56)
heart problems	1538 (11.47)	728 (12.91)	810 (10.42)	11.21	(10.23–12.27)	10.18	(9.05–11.45)	12.41	(10.79–14.23)
chronic lung diseases	1616 (12.05)	688 (12.2)	928 (11.94)	11.82	(10.99–12.71)	11.89	(10.84–13.03)	11.74	(10.45–13.18)
dyslipidemia	1162 (8.66)	535 (9.49)	627 (8.07)	8.86	(7.99–9.81)	8.21	(7.12–9.45)	9.62	(8.28–11.14)
chronic kidney disease	833 (6.21)	324 (5.75)	509 (6.55)	5.96	(5.34–6.64)	6.48	(5.58–7.52)	5.35	(4.57–6.25)
diabetes	743 (5.54)	356 (6.31)	387 (4.98)	5.85	(5.24–6.52)	5.14	(4.45–5.93)	6.67	(5.67–7.83)
Chronic liver disease	521 (3.88)	224 (3.97)	297 (3.82)	3.90	(3.44–4.43)	3.79	(3.23–4.45)	4.03	(3.32–4.90)
asthma	511 (3.81)	206 (3.65)	305 (3.92)	3.69	(3.26–4.17)	3.97	(3.41–4.62)	3.36	(2.75–4.11)
stroke	407 (3.03)	197 (3.49)	210 (2.7)	2.17	(1.83–2.57)	2.00	(1.58–2.52)	2.37	(1.84–3.03)
mental problems	258 (1.92)	111 (1.97)	147 (1.89)	1.78	(1.48–2.14)	1.79	(1.40–2.28)	1.77	(1.34–2.34)
memory-related disease	177 (1.32)	84 (1.49)	93 (1.2)	1.45	(1.16–1.80)	1.43	(1.08–1.89)	1.47	(1.03–2.08)
cancer	132 (0.98)	51 (0.9)	81 (1.04)	0.90	(0.74–1.09)	1.03	(0.82–1.30)	0.74	(0.52–1.04)

Note: *CI* confident interval

Negative binomial regression was employed to investigate variations in multimorbidity. We employed negative binomial regression rather than Poisson regression since the dependent variable’s variance was larger than its mean value. Univariate analyses were performed to examine the association between multimorbidity and each independent variable, respectively. Multivariate negative binomial regression analysis was employed with all covariates introduced. We performed this between-person analysis (Model 1 in Table 3) in order to compare our results in terms of urban-rural disparities with those from prior studies since most of them did not adjust for neighborhood-level variations [21, 24]. Clustered robust standard errors were generated in Model 2 to take individuals nested within neighborhoods into account. Furthermore, we employed the separate logistic regression models with robust standard errors to investigate the association between neighborhoods’ characteristics and the fourteen chronic diseases, respectively (Table 4). Variance inflation factors were calculated to exam collinearity among independent variables. Models’ significance was examined by Pearson chi-square.

**Table 3:** Negative binomial regression analyses of multimorbidity, IRR (95% CI)

Characteristics	Univariate analysis		Model 1		Model 2^a^	
Road type						
unpaved roads	1.00		1.00		1.00	
paved roads	0.88	(0.85–0.92) ^***^	0.87	(0.83–0.91) ^***^	0.86	(0.78–0.95) ^***^
others	0.93	(0.87–0.98) ^***^	0.93	(0.87–0.99) ^**^	0.90	(0.79–1.03)
Urban (vs. rural)	1.04	(1.01–1.08) ^*^	1.05	(1.01–1.09) ^**^	1.02	(0.95–1.10)
Primary care institutions						
0	1.00		1.00		1.00	
1	0.99	(0.95–1.03)	0.99	(0.95–1.03)	1.01	(0.92–1.11)
2	0.99	(0.94–1.04)	1.00	(0.95–1.05)	1.02	(0.92–1.13)
≥ 3	0.90	(0.85–0.96) ^**^	0.94	(0.89–0.99) ^*^	0.94	(0.83–1.06)
Groundwater system (yes vs. no)	1.00	(0.96–1.04)	1.00	(0.96–1.05)	1.02	(0.94–1.11)
Age	1.00	(0.98–1.01)	1.00	(1.00–1.00)	1.00	(1.00–1.00)
Women (vs. men)	1.00	(0.96–1.03)	1.00	(0.96–1.04)	0.99	(0.96–1.03)
Married (vs. others)	0.97	(0.93–1.02)	0.97	(0.93–1.01)	0.97	(0.93–1.02)
Household income						
1st (the poorest)	1.00		1.00		1.00	
2nd	1.03	(0.98–1.07)	1.03	(0.98–1.08)	0.99	(0.95–1.04)
3rd	0.96	(0.91–1.00)	0.95	(0.93–1.00)	0.94	(0.89–0.99) ^*^
4th (the richest)	0.94	(0.89–0.99) ^*^	0.93	(0.89–0.98) ^**^	0.93	(0.88–0.98) ^*^
Education attainment						
illiteracy	1.00		1.00		1.00	
some primary school	1.03	(0.97–1.08)	1.03	(0.97–1.08)	1.03	(0.98–1.08)
primary school	1.01	(0.96–1.06)	1.01	(0.96–1.06)	1.00	(0.95–1.06)
junior school or above	1.03	(0.99–1.07)	1.03	(0.98–1.08)	1.02	(0.97–1.08)
Health care insurance						
uninsured	1.00		1.00		1.00	
RCMI	1.03	(0.96–1.10)	1.03	(0.96–1.11)	1.04	(0.97–1.11)
others	1.04	(0.96–1.12)	1.06	(0.98–1.15)	1.07	(0.99–1.15)
BMI						
< 18.5	1.00		1.00		1.00	
≥ 18.5 and < 25	0.91	(0.85–0.97) ^*^	0.91	(0.85–0.97) ^**^	0.90	(0.84–0.96) ^**^
≥ 25 and < 30	1.15	(1.08–1.24) ^***^	1.16	(1.08–1.25) ^***^	1.14	(1.06–1.22) ^***^
≥ 30	1.36	(1.24–1.50) ^***^	1.37	(1.24–1.50) ^***^	1.33	(1.21–1.46) ^***^
Physical activity						
never	1.00		1.00		1.00	
seldom	1.00	(0.83–1.21)	1.00	(0.83–1.20)	0.96	(0.80–1.15)
a weekly basis	1.09	(0.92–1.29)	1.09	(0.92–1.29)	1.06	(0.90–1.25)
a daily basis	1.10	(1.01–1.20) ^*^	1.08	(0.99–1.18)	1.08	(0.98–1.18)

Note: ^***^ *P* < 0.001; ^**^ *P* < 0.01; ^*^ *P* < 0.05

^a^ standard error was adjusted for clusters within neighborhoods

*IRR* incident rate ratio, *CI* confident interval

**Table 4:** Subgroup logistic regression analyses of the fourteen chronic diseases, OR (95%CI)

characteristics	hypertension	dyslipidemia	diabetes	chronic lung disease	cancer	chronic liver disease	heart problems
unpaved roads	1.00	1.00	1.00	1.00	1.00	1.00	1.00
paved roads	1.00 (0.85–1.18)	1.13 (0.86–1.49)	1.05 (0.83–1.33)	0.76 (0.63–0.93)^**^	0.91 (0.58–1.43)	0.74 (0.55–0.99)^*^	0.86 (0.66–1.11)
others	1.06 (0.84–1.33)	0.75 (0.50–1.12)	0.90 (0.64–1.27)	0.93 (0.71–1.21)	0.80 (0.41–1.55)	0.87 (0.58–1.32)	1.09 (0.76–1.56)
urban (vs. rural)	1.05 (0.92–1.19)	0.95 (0.77–1.17)	1.03 (0.86–1.23)	1.09 (0.93–1.27)	0.80 (0.55–1.16)	1.02 (0.80–1.29)	1.13 (0.93–1.38)
characteristics	stroke	chronic kidney disease	digestive disease	mental problems	memory disease	arthritis or rheumatism	asthma
unpaved roads	1.00	1.00	1.00	1.00	1.00	1.00	1.00
paved roads	1.12 (0.74–1.69)	0.68 (0.51–0.89)^**^	0.82 (0.69–0.97)^*^	0.67 (0.43–1.07)	0.62 (0.38–1.01)	0.69 (0.55–0.87)^**^	0.67 (0.51–0.88)^***^
others	0.86 (0.47–1.57)	0.79 (0.53–1.16)	0.79 (0.63–1.01)	0.79 (0.41–1.51)	0.46 (0.21–1.00)	0.90 (0.65–1.23)	0.66 (0.44–0.98)^*^
urban (vs. rural)	1.09 (0.80–1.49)	0.89 (0.71–1.12)	0.96 (0.84–1.10)	1.26 (0.86–1.84)	1.13 (0.76–1.68)	1.05 (0.88–1.26)	0.97 (0.77–1.23)

Note: ^***^ *P* < 0.001; ^**^ *P* < 0.01; ^*^ *P* < 0.05

As a sensitivity analysis, we performed a multinomial logistic regression with five responses (Y = 0, 1, 2, 3, and > or = 4) with robust standard errors. Results were qualitatively similar to those from negative binomial regression. Statistical analyses were performed with Stata/SE 15.0 (StataCorp, TX, USA). A two-tailed *P* value of less than 0.05 was considered statistically significant.

## Results

### Baseline characteristics

Table 1 presents the baseline characteristics of the study sample. Overall, the present study included 13,413 individuals with 5639 urban residents and 7774 rural residents. First, results suggested that relative to rural areas, urban areas had a higher proportion of individuals living with paved roads (*P* < 0.001). Specifically, 59.09% of the rural residents lived with paved roads, and the figure surged to 74.00% among urban residents. In addition, a higher proportion of urban residents (41.66%) lived with a groundwater system, in comparison to their rural counterparts (20.18%, *P* < 0.001). Last, individual-level attributes, including household income, BMI, and physical activity, varied across urban and rural areas.

### Prevalence of multimorbidity and the fourteen NCDs

According to weighted analyses in Table 2, over 65% of study subjects had at least one NCDs, followed by 19.11% with two NCDs, 9.81% with three NCDs, and around 10% with at least 4 NCDs. The top three NCDs were arthritis or rheumatism (33.08%), hypertension (24.54%), and digestive disease (21.98%). In contrast, cancer, memory-related disease, and mental problems were the least prevalent NCDs. There were 0.90, 1.45, and 1.78% of the subjects self-reporting to have cancer, memory-related disease, and mental problems, respectively. Furthermore, urban subjects were more likely to have hypertension, heart problems, dyslipidemia, and diabetes, whereas rural subjects had a greater probability of asthma (*P* < 0.05). After controlling for CHARLS sampling weights, there existed no urban-rural disparities in the prevalence of each NCD since the 95% CI of urban residents overlapped with that of rural residents across all NCDs.

### H_1_ urban vs. rural disparities

Table 3 presents results from negative binomial analyses. Results suggested that urban residents had a higher probability of more NCDs after controlling for all other covariates (Model 1 in Table 3, IRR = 1.05, 95% CI, 1.01–1.09). Results from Model 2, however, suggested that there existed no urban-rural disparities in the number of NCDs (IRR = 1.02, 95% CI, 0.95–1.02) after taking neighborhood-level variations into account. Results pertaining to urban vs. rural disparities in Model 2 were in line with those from subgroup analyses in Table 4.

### H_2_ Neighborhood’s road types

Results in Table 3 suggest that living with paved roads was associated with fewer NCDs, in comparison with those living with unpaved roads. Specifically, after controlling for all covariates in Model 1, subjects living with paved roads (IRR = 0.87, 95% CI, 0.83–0.91) had a significantly lower incidence rate of more NCDs relative to those living with unpaved roads. Results here were consistent after controlling for neighborhood-level variations in Model 2. Subgroup analyses in Table 4 further suggested that in comparison with those living with unpaved roads, individuals living with paved roads were respectively less likely to have chronic lung disease (OR = 0.76, 95%CI, 0.63–0.92), chronic liver disease (OR = 0.74, 95% CI, 0.55–0.99), chronic kidney disease (OR = 0.67, 95%CI, 0.51–0.89), digestive disease (OR = 0.81, 95%CI, 0.69–0.96), arthritis or rheumatism (OR = 0.69, 95%CI, 0.55–0.87), and asthma (OR = 0.67, 95%CI, 0.50–0.88).

### Neighborhood-level and individual-level covariates

Living in neighborhoods with more primary care institutions was negatively associated with more NCDs (Model 2 in Table 3). Compared with those without primary care institutions in the neighborhood, individuals living with three primary care institutions or more were significantly less likely to have more NCDs (IRR = 0.94, 95% CI, 0.89–0.99). The disparities, however, did not exist after controlling for within-neighborhood variations.

Last, two individual-level attributes, household income, and BMI were associated with NCDs (Model 2 in Table 3). Specifically, compared with the poorest, the richest were less likely to suffer from a higher number of NCDs (IRR = 0.93, 95% CI, 0.88–0.98). Overweight (IRR = 1.14, 95% CI, 1.06–1.22) and obese subjects (IRR = 1.33, 95% CI, 1.21–1.46) respectively had a greater incidence rate of more NCDs compared with those with BMI less than 18.5. Subjects with BMI from18.5 to 25 had a lower incidence rate of more NCDs relative to those BMI less than 18.5, even after controlling for all other covariates (IRR = 0.90, 95%CI, 0.84–0.96).

## Discussion

By employing a national representative sample, the present study depicts the prevalence of multimorbidity and 14 NCDs among Chinese middle-aged and older adults. Although prior studies have focused on the association between neighborhood’s characteristics and residents’ health, the present study expands current understanding by demonstrating the association between neighborhood’s road types and residents’ multimorbidity. In contrast to prior studies [21, 24], we found that there existed no urban-rural disparities in residents’ multimorbidity after controlling for within neighborhoods’ variations.

Our results call for attention directed to non-communicable diseases in China. Relative to other countries, the situation of non-communicable diseases in China is more urgent for its demographic shift and large population. According to our analyses, about 35% of Chinese middle-aged and older adults had comorbidity, whereas the figure in Canada approached 20% among those aged from 45 to 64 [51]. Results show that arthritis or rheumatism was the most prevalent NCDs, followed by hypertension and digestive disease. However, our study only includes observed NCDs, while neglecting those unobserved. One prior study, which analyzed data from the medical questionnaire in CHARLS and included unobserved hypertension, suggested that there were around 40.9% subjects with hypertension [52]. Therefore, it is unwarranted to compare the prevalence of the 14 NCDs due to the exclusion of unobserved NCDs. However, results that the Chinese middle-aged and older adults have a significant proportion with arthritis or rheumatism are consistent with a prior study [53]. Even though arthritis may not contribute to disease burden as considerably as hypertension and diabetes do [54], suffering from arthritis significantly impact older adults’ mobility and quality of life [55, 56].

Our results offer some clues for urban-rural disparities in multimorbidity. Although the urban residents have a greater probability of encountering more NCDs relative to their rural counterparts (univariate analysis and Model 1 in Table 3), the differences appear to be fully explained by within-neighborhood confounding. One possibility is that those with a higher risk of multimorbidity, such as white collars that undertake greater life pressure and live a sedentary lifestyle, tend to be nested in several neighborhoods where residents share similar socioeconomic status [57]. It’s unwarranted to compare our results in Model 2 with prior studies since most of them did not consider within-neighborhood variations. Our between-person results in Model 1 (Table 3) are consistent with results from Korea [21] and Spain [19] but differ from those from Canada [23]. The differences may result from disparities in living environment, occupation, and life behavior between two China and Canada. Given the consistency and heterogeneity, urban-rural disparities in NCDs appear to vary across nations. Therefore, one should be careful to generalize ideas across nations of different social contexts.

Pertaining to the second hypothesis, our results suggest that living with a pave road may be positively associated with residents’ health. On the one hand, results here are in line with findings from prior studies that living with walkable neighborhoods may be associated with the elderly’s social coherence, mental health, and physical activity [25, 58]. On the other hand, we hypothesize that paved roads may link with residents’ access to primary care in China. As upgraded roads could reduce time costs for seeking in-center health care services [38], individuals living with paved roads may be more likely to reduce the progression of certain diseases such as chronic liver disease, chronic lung disease, and chronic kidney disease (subgroup analyses). For arthritis patients and older adults with limited mobility [45], living with paved roads may play a critical role in their access to health care and chronic disease management.

Three implications could be drawn from this study. First, although the prevalence of NCDs among Chinese older population has been extensively discussed, most of these studies focused on hypertension and diabetes [54]. Therefore, we suggest that the local government should pay more attention to the distribution of arthritis or rheumatism-related health resources and health education program. In addition, results suggest that urban-rural disparities may link with within-neighborhood characteristics, and therefore future studies may consider taking within-neighborhood variations into account. Last, our research indicates that the government should consider the local population’s demographic characteristics and health demand, e.g., the proportion of older adults and their mobility. For the population in transportation-disadvantaged regions such as rural areas in China’s Tibet, Xinjiang, and Sichuan province, the distribution of health care resources should consider not only population density but also geographic attributes including land area, road types, and access to public transportation.

This study employed a good representative sample of Chinese middle-aged and older populations with large sample size. We depicted the prevalence of the 14 NCDs and analyzed the variations a function of neighborhood attributes as well as individual characteristics. Robust standard errors were generated in multivariate models to capture within-neighborhood variations.

Despite the strengths, this study has several limitations. First, the cross-sectional study design limited the potential to offer strong evidence for causality, and future longitudinal studies are warranted. Second, this study employed self-reported outcome measures, leading to non-ignorable reporting and recalling bias. As CHARLS only recorded information on the fourteen chronic diseases and observed ones, the present study may underestimate the prevalence of multimorbidity and the fourteen NCDs. Furthermore, our measurement of neighborhood’s walkability, i.e., road type, has not been validated, which made our findings incomparable with those from prior studies. Future studies may incorporate objective and well-received measures that consider a variety of neighborhood characteristics [59, 60]. However, there is little evidence linking neighborhood’s road type with multimorbidity, and the present study addressed the gap. Last, even though multimorbidity has gained increasing attention, it may not be a perfect measure of health status since it fails to capture health variations among patients with the same number of NCDs as well as differences within various combinations of concurrent NCDs.

## Conclusions

Urban vs. rural disparities in multimorbidity link closely with within-neighborhood variations. The improvement in neighborhood-level characteristics, such as road quality, holds promise to alleviate the increasing disease burden of chronic diseases.

## Data Availability

All data are available from CHARLS at: http://charls.pku.edu.cn/.

## Abbreviations

NCDs: Non-communicable chronic diseases
CHARLS: China Health and Retirement Longitudinal Study
BMI: Body mass index
RCMI: Rural cooperative medical insurance
IRR: Incidence rate ratio
OR: Odds ratio
CI: Confident interval
