# Correction to: Neighborhood’s locality, road types, and residents’ multimorbidity: evidence from China’s middle-aged and older adults

**DOI:** 10.1186/s12889-020-09936-3

**Published:** 2020-11-24

**Authors:** Xuexin Yu, Wei Zhang

**Affiliations:** West China Biomedical Big Data Center, West China Hospital, Sichuan University, No.37 Guoxue Alley, Chengdu, 610040 Sichuan China

**Correction to: BMC Public Health 20, 1728 (2020)**

**https://doi.org/10.1186/s12889-020-09876-y**

It was highlighted that in the original article [1] the covariates names in Table 1 were misaligned. This Correction article shows the correct Table 1. The original article has been updated.

**Table 1:** Baseline characteristics of study sample, stratified by urban vs rural settings, n (%)

Characteristic	Total (*N* = 13,413)	Urban (*N* = 5639)	Rural (*N* = 7774)	*P* value
Road type				
unpaved roads	3104 (23.14)	1001 (17.75)	2103 (27.05)	< 0.0001
paved roads	8767 (65.36)	4173 (74.00)	4594 (59.09)	
others	1541 (11.50)	465 (8.54)	1077 (13.85)	
Primary care institutions				
0	3226 (24.05)	1333 (23.64)	1893 (24.35)	0.07
1	5188 (38.68)	2210 (39.19)	2978 (38.31)	
2	3098 (23.10)	1339 (23.75)	1759 (22.63)	
≥ 3	1901 (14.17)	757 (13.42)	1144 (14.72)	
Groundwater system				
no	9495 (70.79)	3290 (58.34)	6205 (79.82)	< 0.0001
yes	3918 (29.21)	2349 (41.66)	1569 (20.18)	
Sex				
men	6431 (47.95)	2660 (47.17)	3771 (48.51)	0.13
women	6982 (52.05)	2979 (52.83)	4003 (51.49)	
Age, years ^a^	59.09 (±10.16)	58.91 (±10.14)	(59.21 (±10.18)	< 0.0001
Marital status				
married	10,741 (80.08)	4475 (79.36)	6266 (80.60)	0.08
other	2672 (19.92)	1164 (20.64)	1508 (19.40)	
Education attainment				
illiteracy	3672 (27.38)	1543 (27.36)	2129 (27.39)	0.67
some primary school	2371 (17.68)	1019 (18.07)	1352 (17.39)	
primary school	2895 (21.58)	1195 (21.19)	1700 (21.87)	
junior school or above	4475 (33.36)	1882 (33.37)	2593 (33.35)	
Household income (quartile)				
1st (the poorest)	3218 (23.99)	1477 (26.19)	1741 (22.40)	< 0.0001
2nd	3466 (25.84)	1363 (24.17)	2103 (27.05)	
3rd	3345 (24.94)	1418 (25.15)	1927 (24.79)	
4th (the richest)	3384 (25.23)	1381 (24.49)	2003 (25.77)	
BMI (kg/m^2^)				
< 18.5	935 (6.97)	358 (6.35)	577 (7.42)	< 0.001
≥ 18.5 and < 25	8362 (62.34)	3449 (61.16)	4913 (63.20)	
≥ 25 and < 30 (overweight)	3462 (25.81)	1532 (27.17)	1930 (24.83)	
≥ 30 (obese)	654 (4.88)	300 (5.32)	354 (4.55)	
Health care insurance				
uninsured	893 (6.66)	375 (6.65)	518 (6.66)	0.14
RCMI	9771 (72.85)	4063 (72.05)	570 (73.42)	
others	2749 (20.50)	1201 (21.30)	1548 (19.91)	
Physical activity				
never	12,686 (94.58)	5144 (91.22)	7542 (97.02)	< 0.0001
seldom	112 (0.84)	75 (1.33)	37 (0.48)	
a weekly basis	129 (0.96)	88 (1.56)	41 (0.53)	
a daily basis	486 (3.62)	332 (5.89)	154 (1.98)	

Note: ^a^mean (± standard deviation)
